# Residential altitude was not independently associated with osteoporosis among long-term Qinghai–Tibetan Plateau residents

**DOI:** 10.3389/fpubh.2026.1844263

**Published:** 2026-05-20

**Authors:** Zhibin Liu, Hao Zhang, Shuzhuo Zhang, Honghao Dai, Junlong Huang, Qianfu Gong, Dajie Suonan, Zhongshu Shan

**Affiliations:** 1 College of Clinical Medicine, Qinghai University, Xining, Qinghai, China; 2 Department of Spine Surgery, Qinghai Provincial People's Hospital, Xining, Qinghai, China; 3 Qinghai Branch, The First Affiliated Hospital of Xi’an Jiaotong University, Xining, Qinghai, China

**Keywords:** 25-hydroxyvitamin D, BMI-related heterogeneity, body mass index, high-altitude residence, Hounsfield units, opportunistic computed tomography, osteoporosis, vertebral trabecular attenuation

## Abstract

**Background:**

The association between long-term high-altitude residence and osteoporosis remains unclear. This study investigated whether body mass index (BMI) and vitamin D status modify the association between residential altitude and dual-energy X-ray absorptiometry (DXA)-defined osteoporosis among long-term residents of the Qinghai–Tibetan Plateau. In resource-limited high-altitude settings, limited access to DXA may hinder timely osteoporosis detection, and opportunistic use of routine computed tomography (CT), together with simple host characteristics such as BMI, may support pragmatic risk stratification.

**Methods:**

A single-centre, hospital-based retrospective case–control study was conducted among residents aged ≥50 years who had undergone non-contrast CT and DXA within 30 days and had lived on the Qinghai–Tibetan Plateau for ≥5 years. Osteoporosis was defined as a DXA T-score ≤ − 2.5, and individuals with osteopenia were excluded to increase phenotypic contrast. Residential altitude (1,500–4,600 m) was geocoded and analysed as a continuous variable per 100 m increment. Pre-specified interaction terms included residential altitude × BMI and residential altitude × 25-hydroxyvitamin D [25(OH)D]. Additional BMI-stratified analyses used 24.0 kg/m^2^ as a clinically interpretable threshold. Exploratory HU-adjusted sensitivity analyses are reported in the Supplementary materials.

**Results:**

Among 377 participants (224 osteoporosis cases and 153 controls with normal bone mineral density), residential altitude was not independently associated with osteoporosis after multivariable adjustment (adjusted odds ratio [aOR] 1.020 per 100 m, 95% CI 0.976–1.065; *p* = 0.374). In the primary interaction model, there was suggestive but inconclusive evidence of BMI-related heterogeneity (altitude × BMI aOR 1.011, 95% CI 0.998–1.023; *p* = 0.087), whereas no interaction with 25(OH)D was observed (*p* = 0.423). BMI-stratified analyses showed directionally different but imprecise altitude–osteoporosis associations.

**Conclusion:**

Residential altitude was not independently associated with osteoporosis in this hospital-based case–control study of long-term plateau residents. The data suggested possible BMI-related heterogeneity, but the evidence was inconclusive and should be interpreted cautiously. These findings are hypothesis-generating and may inform future opportunistic bone health assessment in resource-limited high-altitude settings.

## 1 Introduction

Osteoporosis and fragility fractures are recognized as major contributors to disability, reduced functional independence, and increased healthcare use worldwide, particularly in ageing populations (1, 2). In China, the burden associated with osteoporosis is considerable, and it may be further intensified in plateau regions, where distinctive environmental exposures are present and access to specialized screening and preventive services remains limited (3). The identification of setting-specific determinants of skeletal fragility in these areas is therefore needed, both to improve etiological understanding and to support more accurate clinical risk stratification.

Residential altitude has increasingly been recognized as a possible environmental factor in bone health, although its role is still not fully understood (4). Living at high altitude involves long-term exposure to hypobaric hypoxia and is often accompanied by cold temperatures, differences in ultraviolet radiation, dietary limitations, and uneven access to healthcare (5, 6). These conditions may influence bone remodelling through several biological mechanisms. Evidence from both experimental and clinical studies suggests that hypoxia-related signalling may interfere with the normal balance between osteoblast and osteoclast activity (7). At the same time, oxidative stress, chronic inflammatory responses, and disruption of calcium-phosphate balance may further compromise skeletal metabolism. Differences in vitamin D status, body composition, and gut microbial profiles across altitude settings may also play a part in skeletal adaptation (8–11). Taken together, these observations suggest that residence at high altitude may plausibly be linked to an increased risk of osteoporosis.

Even so, epidemiological findings have remained mixed. Some studies have reported lower bone mineral density or greater skeletal fragility in high-altitude populations, whereas others have not found a clear reduction in bone density despite observing altered mineral-regulating hormones and more frequent vitamin D deficiency (12, 13). This lack of consistency suggests that the skeletal effects of altitude are unlikely to be uniform and may vary according to individual metabolic and nutritional characteristics. Among the factors that may shape this relationship, adiposity deserves particular attention.

In lowland populations, a higher body mass index (BMI) is generally associated with greater bone mineral density and a lower risk of fracture (14, 15). This pattern is often explained by increased mechanical loading and higher peripheral oestrogen production from adipose tissue (16, 17). However, such a protective effect may not persist under chronic hypoxic conditions. Obesity is associated with adipose tissue hypoxia, low-grade inflammation, and abnormal adipokine signalling, all of which may have adverse effects on bone remodelling (18, 19). Meanwhile, experimental and population-based studies suggest that chronic high-altitude or hypobaric hypoxia can impair bone mass, although epidemiological findings across plateau settings are not entirely consistent (4, 10, 20, 21). It is therefore possible that, in high-altitude environments, the metabolic disadvantages associated with excess adiposity could reduce or even outweigh the skeletal benefits that are usually linked to a higher BMI. This raises the possibility of BMI-related heterogeneity in altitude-associated osteoporosis risk, meaning that overweight or obesity may not necessarily provide the expected protection against osteoporosis in plateau populations; however, direct evidence for this interaction in humans remains limited (21). Serum 25-hydroxyvitamin D [25(OH)D] may also influence this association, as it reflects mineral-regulatory capacity in settings where abundant sunlight exposure, dietary intake, and physiological adaptation do not necessarily translate into adequate vitamin D status (22, 23).

This question also has practical importance from a clinical perspective. Although dual-energy X-ray absorptiometry (DXA) remains the reference standard for densitometric diagnosis of osteoporosis (24), access to DXA is limited in many resource-constrained settings (25, 26). Opportunistic measurement of vertebral trabecular attenuation on computed tomography, expressed in Hounsfield units (HU), offers a useful complementary screening approach for skeletal assessment when CT scans have already been obtained for other indications (27–29). Because this strategy uses existing imaging, it can help identify individuals at high risk of low bone mineral density without additional radiation exposure or dedicated osteoporosis imaging (30). This approach may therefore be especially relevant in plateau regions and other geographically remote settings where scalable case-finding strategies are needed, although region-specific validation remains important before universal HU thresholds are applied (28, 30).

Beyond its etiologic relevance, this question also has potential public health importance in plateau regions. In many high-altitude and geographically remote settings, access to standard osteoporosis screening with DXA is often limited by cost, equipment availability, and referral barriers. At the same time, routine CT examinations are increasingly available in clinical practice and may offer an opportunistic approach to bone health assessment. Identifying whether residential altitude is associated with osteoporosis risk, and whether this association varies by readily available host characteristics such as BMI, may therefore help inform pragmatic risk stratification and targeted screening strategies in underserved plateau populations. Accordingly, this study aimed not only to examine the association between residential altitude and osteoporosis among plateau residents, but also to explore whether BMI modifies this relationship in a manner that could be relevant for public health screening in resource-constrained high-altitude settings.

## 2 Materials and methods

### 2.1 Study design and ethics approval

A single-centre, hospital-based retrospective case–control study was conducted to investigate the relationship between current residential altitude and DXA-defined osteoporosis. Possible modification of this association by body mass index (BMI) and serum 25-hydroxyvitamin D [25(OH)D] was further evaluated. An extreme-phenotype sampling strategy was applied, with the analytic sample restricted to osteoporosis cases and controls with normal bone mineral density; individuals with osteopenia were excluded. By increasing phenotypic contrast, this strategy was intended to enhance the efficiency of identifying potential interaction effects in regression models. Clinical and imaging information was retrieved from the electronic medical record (EMR) system and the radiology picture archiving and communication system (PACS) of Qinghai Provincial People’s Hospital. Data from clinical encounters and imaging examinations performed between January 1, 2022, and December 31, 2025, were included. The study was approved by the Institutional Review Board of Qinghai Provincial People’s Hospital (Approval No. (2026)-089–01) and conducted in accordance with the Declaration of Helsinki. The requirement for informed consent was waived because of the retrospective design and the use of de-identified data (31, 32).

Although extreme-phenotype sampling improves phenotypic contrast and facilitates the detection of heterogeneity, effect-size estimates—particularly odds ratios—should not be interpreted as directly generalisable to community-based populations. These estimates also do not represent absolute risk differences or community-level risk analogues. No *a priori* sample-size calculation was performed; all consecutive eligible participants identified within the prespecified study window were included in the analytic sample. Given the final sample size (224 cases and 153 normal-BMD controls), the study was better powered for main effects than for interaction terms, and interaction estimates were therefore expected to have limited precision.

### 2.2 Participants: inclusion and exclusion criteria

Participants were consecutively identified from hospital databases to minimise potential selection bias arising from investigator discretion. Eligibility criteria were defined as follows: (1) age ≥50 years; (2) availability of both a routine non-contrast CT scan (chest and/or abdomen) and a DXA examination of the lumbar spine (L1–L4) and/or femoral neck obtained during routine clinical care; (3) a CT-to-DXA interval ≤30 days to ensure temporal comparability between skeletal phenotype and densitometric status; and (4) current residence on the Qinghai–Tibetan Plateau for ≥5 years, determined from the current residential address documented in hospital records and, when explicitly available, from residence-duration information in the medical record. The prespecified 5-year criterion was adopted for pragmatic reasons to enrich the sample for sustained altitude exposure and reduce the inclusion of recent migrants; it should not be interpreted as a biologically validated threshold.

Exclusion criteria were prespecified to maintain measurement validity and minimise major secondary causes of bone loss. First, CT images that did not allow valid assessment of vertebral trabecular bone were excluded, including those with moderate or severe vertebral compression fractures, major structural abnormalities, focal lesions, or severe imaging artefacts. When feasible, a predefined vertebral substitution sequence (L1 → L2 → L3 → T12) was applied to maintain measurement standardisation. Second, participants with uncontrolled systemic conditions affecting bone or mineral metabolism were excluded, including uncontrolled hyperparathyroidism, uncontrolled hyperthyroidism, Cushing syndrome, advanced chronic kidney disease with renal osteodystrophy, or active rheumatoid arthritis. Third, individuals who had received prescription anti-osteoporosis medications before the baseline CT examination were excluded, including bisphosphonates, denosumab, selective oestrogen receptor modulators, or anabolic agents. Fourth, participants with missing non-imputable core variables required to define eligibility or the primary exposure/outcome were excluded, such as unavailable DXA classification, missing residential address for geocoding, or unavailable CT data for vertebral assessment.

**Figure 1:**
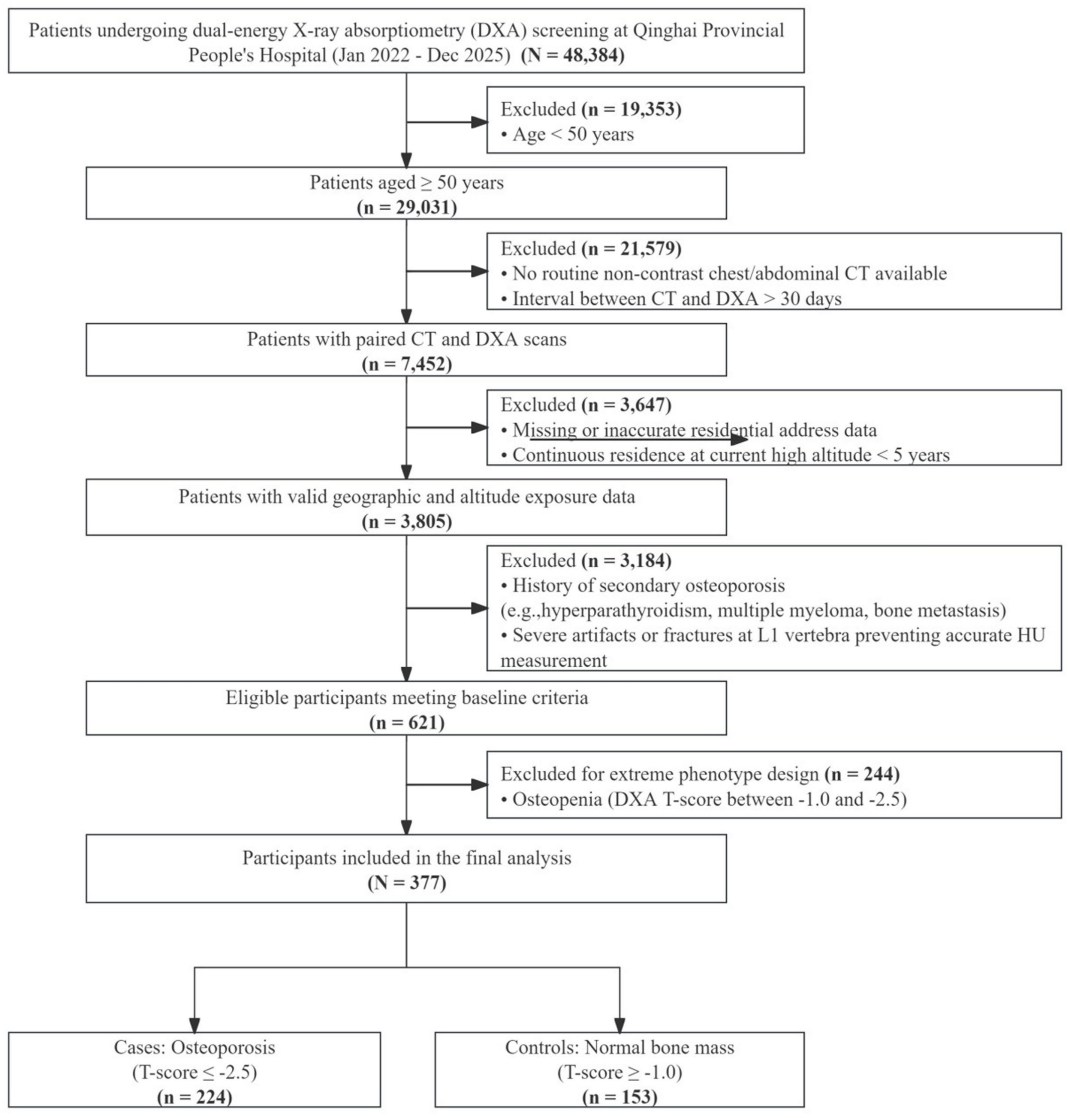
Flowchart of participant selection. To enhance statistical power for the evaluation of potential modifying effects, an extreme-phenotype sampling design was adopted. Patients with osteopenia (T-score between −1.0 and −2.5) were therefore excluded, which increased phenotypic separation between osteoporosis cases and controls with normal bone mineral density.

Missing values in laboratory covariates were addressed using multiple imputation rather than excluding participants. Controls were primarily selected from individuals undergoing routine annual health examinations, opportunistic screening, or follow-up imaging for non-metabolic indications. Osteoporosis cases, by contrast, were more often identified through routine clinical referral pathways for bone health assessment or related care. Specialty clinic populations were avoided where possible to reduce hospital selection artefacts; however, these differing entry pathways may still have introduced selection differences between cases and controls. The participant selection process is summarised in Figure 1.

### 2.3 Measurements and definitions

#### 2.3.1 Outcome: DXA-defined osteoporosis

The primary outcome was osteoporosis status, defined according to the World Health Organization operational criteria based on DXA T-scores (33, 34). Bone mineral density (BMD) was measured using a Lunar iDXA densitometer (GE Healthcare, Madison, WI, USA) with enCORE 2011 software (version 13.60.033). Lumbar spine and proximal femur scans were acquired and analyzed in accordance with the recommendations of the International Society for Clinical Densitometry (ISCD). T-scores were interpreted using the Chinese reference database embedded in enCORE 2011 software at our centre, and osteoporosis was defined as a T-score of ≤ − 2.5 at the lumbar spine or femoral neck. When both skeletal sites were available, classification was based on the lowest eligible T-score. Vertebrae with obvious structural abnormalities, severe degenerative changes, fracture, metallic instrumentation, or other artifacts that could invalidate measurement were excluded from lumbar spine interpretation in accordance with ISCD guidance (34).

#### 2.3.2 Primary exposure: residential altitude

**Figure 2:**
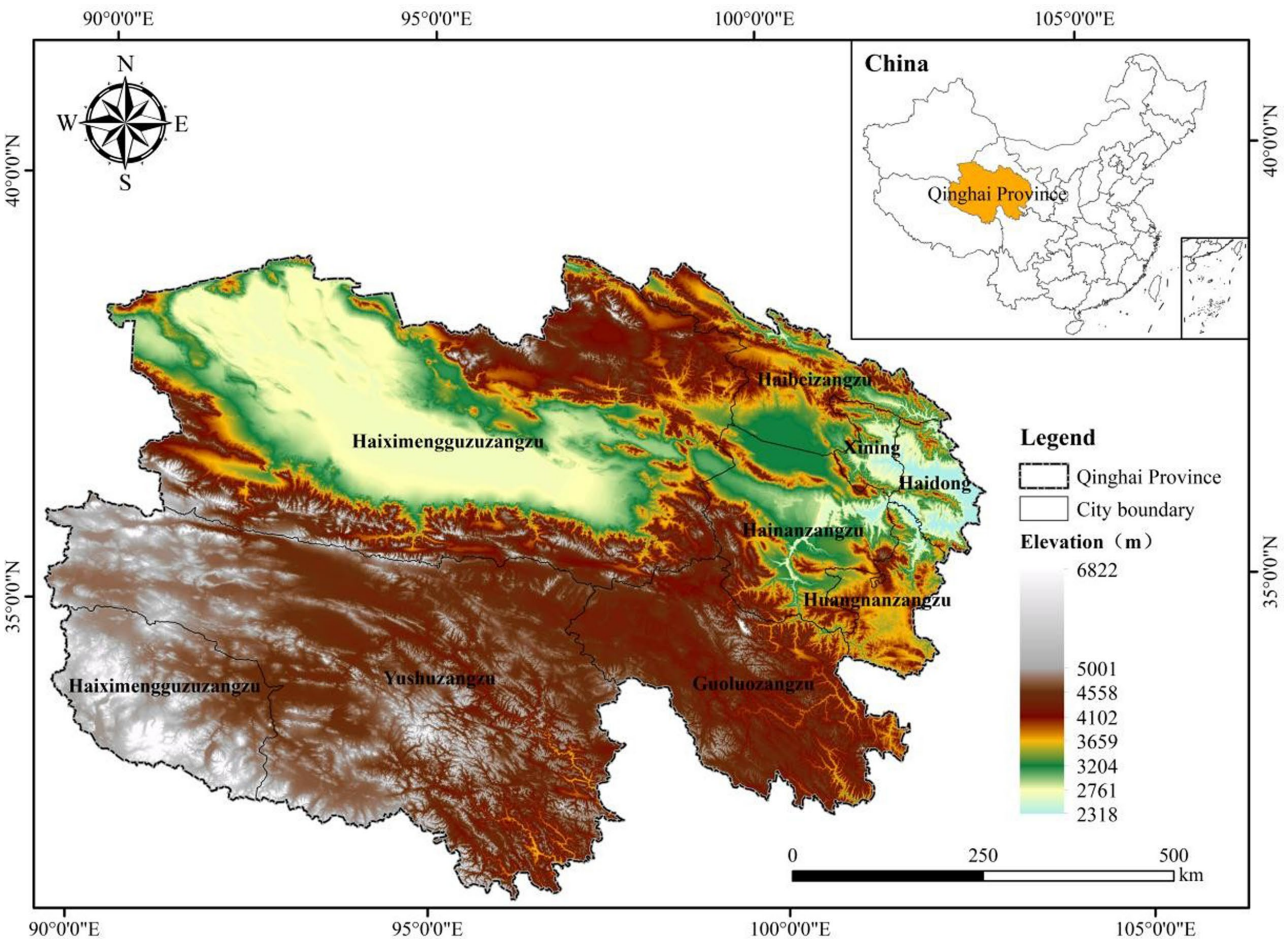
Topographic elevation map of Qinghai Province, China. The map shows the elevation pattern of Qinghai Province across the northeastern Tibetan Plateau, highlighting marked spatial variation in terrain, including the low-lying Qaidam Basin in the northwest and the Huangshui Valley near Xining. Elevation is represented by hypsometric tints. Provincial and prefecture-level city boundaries are displayed, and the inset map indicates the location of Qinghai within China. This geographic context supports the assessment of residential altitude exposure in the study population.

Current residential altitude was defined as the primary environmental exposure. It was derived through geographic information system-based geocoding of each participant’s current residential address as recorded in hospital records. Each address was geocoded using a map application programming interface to obtain elevation above sea level (metres). Current residential altitude was modelled as a continuous variable and expressed per 100 m increase to improve the interpretability of odds ratios and enhance comparability across models. In the analytic cohort, current residential altitude ranged from approximately 1,500 to 4,600 m, thereby capturing meaningful variation in residential elevation among plateau residents. Because detailed lifetime migration history, seasonal mobility, occupational altitude exposure, and other time-varying exposure histories were not available, current residential altitude was interpreted as a pragmatic proxy for sustained altitude exposure rather than a direct measure of cumulative lifetime exposure. Accordingly, some exposure misclassification and residual confounding remain possible, particularly among older adults who may have moved across altitude strata over time (Figure 2).

#### 2.3.3 Effect modifiers and covariates

Potential effect modification was examined primarily for BMI and serum 25(OH)D, while additional covariates were selected according to their clinical relevance to osteoporosis and mineral metabolism.

BMI (kg/m^2^) was calculated from measured height and weight. In interaction analyses, BMI was modeled as a continuous variable. In stratified analyses, it was further dichotomized at 24.0 kg/m^2^ based on the Chinese adult threshold for overweight, thereby facilitating clinical interpretation (35).

Opportunistic attenuation measurements were derived from non-contrast CT scans acquired on a Revolution CT scanner (GE Medical Systems, LLC, Waukesha, WI, USA). The CT acquisition parameters included a tube voltage of 120 kVp, automatic tube current modulation, and a reconstructed slice thickness of 5 mm. Vertebral attenuation was assessed at the L1 vertebral body whenever evaluable (27, 36). If L1 was unsuitable because of focal lesion, fracture, severe artifact, or other technical limitations, the next available vertebra was used in the following order: L2, L3, and T12. For each vertebra, oval regions of interest were placed within the trabecular bone on three axial slices: just inferior to the superior endplate, at the mid-vertebral level, and just superior to the inferior endplate. Regions of interest were placed carefully to avoid the cortical shell, basivertebral venous plexus, focal sclerosis, Schmorl’s nodes, fractures, and other visible abnormalities. The mean of the three measurements was used as the vertebral attenuation value in Hounsfield units (HU). Measurements were independently performed by two trained readers, and inter-reader agreement was excellent, with an intraclass correlation coefficient (ICC) of 0.991 (37).

Fasting morning blood samples were obtained to measure serum 25(OH)D, albumin-corrected calcium, phosphate, magnesium, alkaline phosphatase, sodium, and related biochemical parameters. These measurements were performed using standard hospital laboratory procedures. Albumin-corrected calcium was calculated with the laboratory’s validated correction equation.

A history of prior vertebral fragility fracture was ascertained through medical record review and required imaging confirmation by radiography, CT, or MRI. When applicable, fracture severity was graded using the Genant semiquantitative method to standardise ascertainment across imaging modalities (38).

#### 2.3.4 *A priori* definition of vitamin D deficiency

Vitamin D deficiency was defined *a priori* as serum 25(OH)D < 20 ng/mL (50 nmol/L), consistent with established clinical guideline thresholds (39). Serum 25(OH)D was retained as a continuous covariate in regression models to preserve information.

### 2.4 Statistical analysis

#### 2.4.1 Descriptive statistics

Statistical analyses were performed using IBM SPSS Statistics version 27.0 and R version 4.5.2. Continuous variables are presented as median (interquartile range, IQR). Differences between cases and controls were evaluated using the Mann–Whitney U test because normality was not assumed. Categorical variables are presented as n (%). Group differences were assessed using the chi-square test or Fisher’s exact test, as appropriate.

#### 2.4.2 Handling of missing data

Missing data were observed primarily in laboratory variables, whereas core variables—including age, sex, BMI, residential altitude, DXA classification, and L1 HU—were complete. Assuming a missing-at-random mechanism, missing laboratory values were handled using multiple imputation by chained equations (MICE) (40, 41). Twenty imputed datasets were generated. Regression coefficients and standard errors were pooled across the imputed datasets using Rubin’s rules (42). Although earlier recommendations suggested that a small number of imputations may be acceptable when the fraction of missing information is not high, more recent guidance often recommends a larger number of imputations to improve the stability of standard errors and *p*-values; therefore, the present results should be interpreted with appropriate caution.

#### 2.4.3 Univariable logistic regression

Each candidate predictor was first evaluated using univariable binary logistic regression with osteoporosis status (yes/no) as the dependent variable. Crude odds ratios (ORs) and 95% confidence intervals (CIs) were estimated. Covariate inclusion in multivariable models was guided by prior clinical knowledge and plausible confounding structures rather than by univariable statistical significance alone.

#### 2.4.4 Multivariable model specification and stepwise conceptual build

Multivariable logistic regression analyses were conducted using three prespecified models.

Model 1 was specified as the main-effects model and included age, sex, BMI, residential altitude, serum 25(OH)D, prior vertebral fracture history, serum sodium, and albumin-corrected calcium.

Model 2 extended Model 1 by adding the prespecified interaction terms residential altitude × BMI and residential altitude × 25(OH)D. These terms were used to formally test effect modification on the multiplicative scale.

Model 3 additionally adjusted for L1 trabecular HU. Because HU may represent a proximal skeletal phenotype closely related to the outcome and may lie on the pathway between altitude exposure and osteoporosis status, Model 3 was prespecified as an exploratory sensitivity analysis rather than the primary inferential model; full Model 3 results are provided in Supplementary materials.

#### 2.4.5 Multicollinearity control and scaling for interpretability

To reduce multicollinearity between continuous predictors and their interaction terms and to improve interpretability, continuous variables were mean-centred before interaction modelling. Residential altitude was expressed per 100 m increase, and HU per 10 HU increase, before centring. Variance inflation factors were examined after centring to assess potential multicollinearity. BMI was analysed as a continuous variable in the interaction models. The threshold of 24.0 kg/m^2^ was used only for stratified analyses.

#### 2.4.6 Effect-modification testing and BMI-stratified analyses

Effect modification was assessed by examining the statistical significance of the residential altitude × BMI and residential altitude × 25(OH)D interaction terms in Model 2. Two-sided tests with *α* = 0.05 were used. To facilitate clinical interpretation, prespecified BMI-stratified multivariable analyses were additionally conducted for the primary interaction model (Model 2). A threshold of 24.0 kg/m^2^ was used to define lower-BMI (<24 kg/m^2^; non-overweight by the Chinese adult threshold) and higher-BMI (≥24 kg/m^2^) subgroups. Covariate adjustment within each stratum followed the same framework as the primary model to maintain comparability across strata. Because formal interaction testing in a sample of this size may have limited precision, these stratified analyses were prespecified as hypothesis-generating aids to interpretation and were not intended to establish definitive subgroup effects in the absence of clear interaction evidence. Additional HU-adjusted stratified sensitivity analyses are provided in Supplementary materials.

#### 2.4.7 Marginal effects visualization

**Figure 3:**
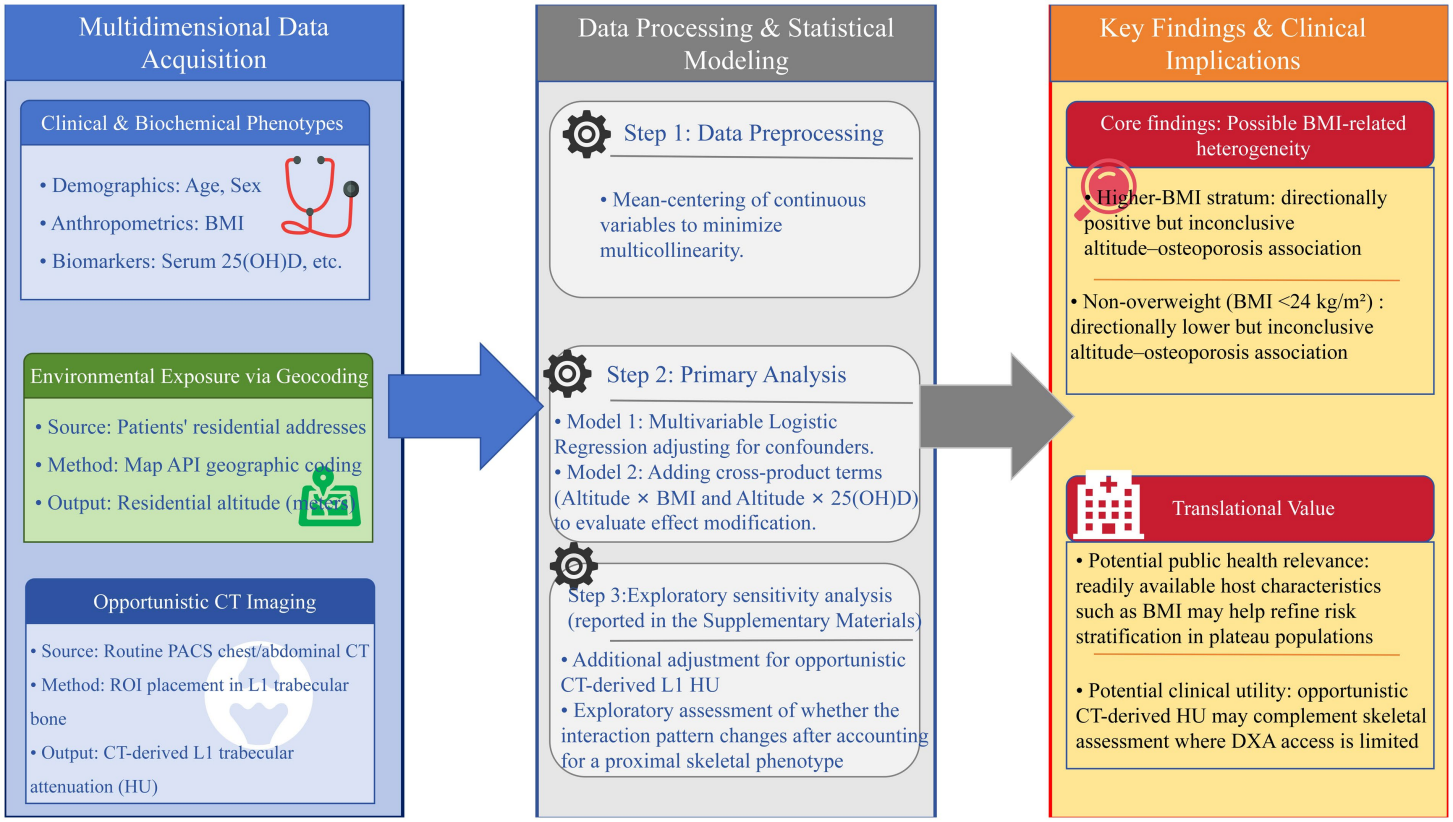
Conceptual framework and study workflow. The study integrated multidimensional data acquisition, including clinical and biochemical phenotypes, environmental exposure assessment through geocoded residential altitude, and opportunistic CT-derived trabecular bone attenuation at L1 (Hounsfield units, HU). Statistical analysis followed a stepwise multivariable logistic regression framework: Model 1 estimated adjusted main effects, Model 2 tested prespecified interaction terms, and Model 3 performed an exploratory sensitivity analysis incorporating L1 HU. The figure summarizes the hypothesized altitude–BMI interaction and the analytic strategy used to evaluate possible BMI-related heterogeneity in altitude-associated osteoporosis risk. Any stratum-specific patterns shown should be interpreted cautiously and as hypothesis-generating. The lower-BMI group is defined as BMI < 24 kg/m^2^ (non-overweight), not underweight.

To provide a more complete view of the joint relationship between BMI and altitude than could be conveyed by a single interaction term alone, model-based marginal effects were visualized in two complementary ways. First, a predicted-probability heatmap was constructed to display the estimated risk of osteoporosis across the combined distribution of BMI and residential altitude, while serum 25(OH)D was held at the sample median. Second, conditional odds-ratio curves were plotted to show how the altitude-associated odds ratio per 100 m increase varied continuously across BMI. To limit instability related to extrapolation, BMI was restricted to the 5th–95th percentile range of the observed sample. Model-based marginal effects for the primary interaction model (Model 2) were visualized in the main manuscript. Corresponding HU-adjusted sensitivity visualizations are provided in Supplementary materials. The overall conceptual framework and analytic workflow of the study are summarised in Figure 3.

## 3 Results

### 3.1 Baseline characteristics of the study population

A total of 377 long-term residents of the Qinghai–Tibetan Plateau were included in the final analytic sample, including 224 osteoporosis cases and 153 normal-BMD controls (Figure 1). Residential altitude ranged from approximately 1,500 to 4,600 m. The median age of the overall cohort was 68.0 years (IQR, 17.0), 264 participants (70.0%) were female, the median BMI was 23.4 kg/m^2^ (IQR, 4.9), and the median residential altitude was 2,835 m (IQR, 1,337) (Table 1).

**Table 1:** Baseline characteristics of the study population by osteoporosis status.

Variable	Overall (n = 377)	Osteoporosis (n = 224)	Normal BMD (n = 153)	*p*-value
Age, years, median (IQR)	68.00 (17.00)	73.00 (12.00)	61.00 (16.00)	**<0.001**
Female, n (%)	264 (70.0%)	195 (87.1%)	69 (45.1%)	**<0.001**
BMI, kg/m^2^, median (IQR)	23.40 (4.90)	22.55 (4.30)	25.10 (4.50)	**<0.001**
Residential altitude, m, median (IQR)	2,835 (1337)	2,835 (1337)	2,800 (1458)	0.501
Opportunistic CT trabecular attenuation (L1 HU), median (IQR)	76.83 (74.16)	54.78 (36.16)	131.97 (50.64)	**<0.001**
Corrected calcium, mmol/L, median (IQR)	2.28 (0.12)	2.29 (0.13)	2.27 (0.13)	0.058
25(OH)D, ng/mL, median (IQR)	12.76 (10.20)	11.20 (10.02)	14.62 (9.66)	0.256
Potassium, mmol/L, median (IQR)	3.88 (0.51)	3.88 (0.53)	3.86 (0.47)	0.873
Sodium, mmol/L, median (IQR)	140.00 (3.00)	140.00 (3.00)	139.00 (3.00)	**0.079**
Magnesium, mmol/L, median (IQR)	0.84 (0.10)	0.84 (0.11)	0.84 (0.10)	0.664
Phosphate, mmol/L, median (IQR)	1.11 (0.27)	1.1 (0.26)	1.10 (0.28)	0.326
Chloride, mmol/L, median (IQR)	106.70 (3.60)	106.80 (3.80)	106.55 (3.03)	0.420
Prior vertebral fracture, n (%)	165 (43.8%)	148 (66.1%)	17 (11.1%)	**<0.001**

Continuous variables are presented as median (interquartile range, IQR), and categorical variables as *n* (%). *p-*values were calculated using the Mann–Whitney U test for continuous variables and the chi-square test or Fisher’s exact test, as appropriate, for categorical variables. Osteoporosis was defined as a DXA T-score ≤ − 2.5 at the lumbar spine (L1–L4) and/or femoral neck, and normal BMD as a T-score ≥ − 1.0; participants with osteopenia were excluded by design. Descriptive statistics shown in this table are based on observed data for each variable. Bold values indicate statistically significant differences (two-sided test, *p* < 0.05).

Compared with controls, participants with osteoporosis were older (median, 73.0 vs. 61.0 years; *p <* 0.001), were more often female (87.1% vs. 45.1%; *p <* 0.001), and had a lower BMI (median, 22.55 vs. 25.10 kg/m^2^; *p <* 0.001). Residential altitude did not differ significantly between the two groups (median, 2,835 vs. 2,800 m; *p* = 0.501).

Clear between-group differences were observed in skeletal phenotypes. The median opportunistic CT-derived L1 trabecular attenuation was substantially lower in the osteoporosis group than in the normal-BMD group (54.78 vs. 131.97 HU; *p <* 0.001). A history of prior vertebral fragility fracture was also more common among cases than controls (66.1% vs. 11.1%; *p <* 0.001). No statistically significant between-group differences were observed for corrected calcium (*p* = 0.058), serum 25(OH)D (*p* = 0.256), potassium (*p* = 0.873), sodium (*p* = 0.079), magnesium (*p* = 0.664), phosphate (*p* = 0.326), or chloride (*p* = 0.420). Missing data were mainly confined to laboratory variables, whereas core exposure, outcome, anthropometric, and HU variables were complete. All regression analyses reported below were pooled across 20 multiply imputed datasets (Supplementary Table S1).

### 3.2 Univariable logistic regression results

Results of the univariable logistic regression analyses are presented in Table 2. Older age, female sex, lower BMI, lower L1 trabecular HU, and a history of prior vertebral fracture were each strongly associated with higher odds of osteoporosis. In contrast, residential altitude showed no evidence of a significant unadjusted main effect.

**Table 2:** Univariable logistic regression analyses for osteoporosis.

Predictor	Unit/contrast	OR (95% CI)	*P*-value
Age	per +1 year	1.096 (1.070–1.122)	**<0.001**
Female sex	female vs. male	8.186 (4.947–13.544)	**<0.001**
BMI	per +1 kg/m^2^	0.799 (0.746–0.855)	**<0.001**
Residential altitude	per +100 m	0.988 (0.960–1.017)	0.417
L1 trabecular HU	per +10 HU	0.456 (0.388–0.537)	**<0.001**
Corrected calcium	per +1 mmol/L	7.552 (1.098–51.928)	0.040
25(OH)D	per +1 ng/mL	0.985 (0.963–1.008)	0.181
Potassium	per +1 mmol/L	0.946 (0.558–1.603)	0.836
Sodium	per +1 mmol/L	1.090 (1.011–1.176)	**0.025**
Magnesium	per +1 mmol/L	1.737 (0.201–14.986)	0.616
Phosphate	per +1 mmol/L	1.941 (0.599–6.291)	0.269
Chloride	per +1 mmol/L	1.029 (0.967–1.095)	0.367
Prior vertebral fracture	yes vs. no	15.579 (8.766–27.688)	**<0.001**

Odds ratios (ORs) and 95% confidence intervals (CIs) were estimated from separate univariable binary logistic regression models with osteoporosis status (yes/no) as the outcome. Continuous predictors were modelled per unit increase as indicated in the “Unit/contrast” column: age per 1 year, BMI per 1 kg/m^2^, residential altitude per 100 m, L1 trabecular HU per 10 HU, and laboratory variables per 1 mmol/L or 1 ng/mL, as applicable. Reported estimates are pooled across 20 multiply imputed datasets for variables subject to missingness. *p-*values are two-sided. The crude corrected-calcium estimate should be interpreted cautiously because its wide confidence interval suggests limited precision and possible sparse-data instability. Bold values indicate statistically significant differences (two-sided test, *p* < 0.05).

Specifically, the odds ratio for osteoporosis was 1.096 per one-year increase in age (95% CI, 1.070–1.122; *p <* 0.001) and 8.186 for females compared with males (95% CI, 4.947–13.544; *p <* 0.001). BMI was inversely associated with osteoporosis (OR, 0.799 per 1 kg/m^2^; 95% CI, 0.746–0.855; *p <* 0.001). A similar inverse association was observed for L1 trabecular attenuation (OR, 0.456 per 10 HU; 95% CI, 0.388–0.537; *p <* 0.001). Prior vertebral fracture was also strongly associated with osteoporosis (OR, 15.579; 95% CI, 8.766–27.688; *p <* 0.001).

Residential altitude was not associated with osteoporosis in the univariable model (OR, 0.988 per 100 m; 95% CI, 0.960–1.017; *p* = 0.417). Among biochemical variables, corrected calcium (OR, 7.552; 95% CI, 1.098–51.928; *p* = 0.040) and sodium (OR, 1.090; 95% CI, 1.011–1.176; *p* = 0.025) were positively associated with osteoporosis. No statistically significant associations were observed for serum 25(OH)D (*p* = 0.181), potassium (*p* = 0.836), magnesium (*p* = 0.616), phosphate (*p* = 0.269), or chloride (*p* = 0.367). Inter-reader agreement for L1 trabecular HU measurement was excellent (single-measure ICC = 0.991; 95% CI, 0.988–0.993). Bland–Altman analysis showed minimal systematic bias (Supplementary Figure S1 and Supplementary Table S2).

### 3.3 Main-effects multivariable model (model 1)

After adjustment for prespecified covariates in Model 1 (Table 3), osteoporosis remained independently associated with older age, female sex, lower BMI, and prior vertebral fracture history, whereas residential altitude was not significantly associated with the outcome. Specifically, the adjusted odds ratio (aOR) for osteoporosis was 1.099 per one-year increase in age (95% CI, 1.064–1.136; *p <* 0.001). The aOR was 11.400 for females compared with males (95% CI, 5.442–23.878; *p <* 0.001). BMI was inversely associated with osteoporosis (aOR, 0.792 per 1 kg/m^2^ increase; 95% CI, 0.724–0.867; *p <* 0.001). A strong association was also observed for prior vertebral fracture (aOR, 10.808; 95% CI, 5.406–21.610; *p <* 0.001). Residential altitude remained non-significant after adjustment (aOR, 1.020 per 100 m; 95% CI, 0.976–1.065; *p* = 0.374). Sodium (aOR, 1.061; 95% CI, 0.958–1.174; *p* = 0.256), serum 25(OH)D (aOR, 0.999; 95% CI, 0.963–1.036; *p* = 0.962), and corrected calcium (aOR, 4.258; 95% CI, 0.489–37.077; *p* = 0.189) were not statistically significant in the adjusted main-effects model.

**Table 3:** Main-effects and primary interaction models for osteoporosis (models 1–2).

Predictor	Coding/unit	Model 1 aOR (95% CI)	*p-*value	Model 2 aOR (95% CI)	*p-*value
Main effects and covariates					
Age	per +1 year	1.099 (1.064–1.136)	**<0.001**	1.099 (1.064–1.136)	**<0.001**
Female sex	female vs. male	11.400 (5.442–23.878)	**<0.001**	11.406 (5.436–23.933)	**<0.001**
BMI	per +1 kg/m^2^	0.792 (0.724–0.867)	**<0.001**	0.774 (0.704–0.851)	**<0.001**
Residential altitude	per +100 m	1.020 (0.976–1.065)	0.374	1.016 (0.972–1.063)	0.474
Prior vertebral fracture	yes vs. no	10.808 (5.406–21.610)	**<0.001**	12.001 (5.857–24.591)	**<0.001**
Sodium	per +1 mmol/L	1.061 (0.958–1.174)	0.256	1.062 (0.959–1.175)	0.246
25(OH)D	per +1 ng/mL	0.999 (0.963–1.036)	0.962	0.999 (0.961–1.038)	0.944
Corrected calcium	per +1 mmol/L	4.258 (0.489–37.077)	0.189	4.113 (0.488–34.661)	0.193
Interaction terms					
Altitude × BMI	interaction term	—	—	1.011 (0.998–1.023)	0.087
Altitude × 25(OH)D	interaction term	—	—	0.998 (0.992–1.003)	0.423

Adjusted odds ratios (aORs) and 95% confidence intervals (CIs) were estimated using multivariable binary logistic regression with osteoporosis status (yes/no) as the outcome. Model 1 included age, sex, BMI, residential altitude, prior vertebral fracture, sodium, serum 25(OH)D, and albumin-corrected calcium. Model 2 additionally included the prespecified interaction terms residential altitude × BMI and residential altitude × 25(OH)D. Continuous predictors were modelled per unit increase as indicated in the “Coding/unit” column. Residential altitude was scaled per 100 m. To reduce collinearity, continuous variables entering interaction terms were mean-centred before construction of cross-product terms. Reported estimates are pooled across 20 multiply imputed datasets where applicable. *p-*values are two-sided Wald test *p-*values for the corresponding coefficients; for the rows “Altitude × BMI” and “Altitude × 25(OH)D”, the *p-*values represent formal tests of multiplicative interaction. Bold values indicate statistically significant differences (two-sided test, *p* < 0.05).

### 3.4 Multivariable interaction model (model 2)

When the prespecified interaction terms were introduced in Model 2 (Table 3), there was borderline evidence that the association between residential altitude and osteoporosis varied according to BMI. No comparable interaction was observed for serum 25(OH)D.

In Model 2, the residential altitude × BMI interaction term had an estimated odds ratio of 1.011 (95% CI, 0.998–1.023; *p* = 0.087). The residential altitude × 25(OH)D interaction term was 0.998 (95% CI, 0.992–1.003; *p* = 0.423). The main effect of residential altitude remained non-significant (aOR, 1.016 per 100 m; 95% CI, 0.972–1.063; *p* = 0.474). The adjusted associations for age (aOR, 1.099; 95% CI, 1.064–1.136; *p <* 0.001), female sex (aOR, 11.406; 95% CI, 5.436–23.933; *p <* 0.001), BMI (aOR, 0.774; 95% CI, 0.704–0.851; *p <* 0.001), and prior vertebral fracture (aOR, 12.001; 95% CI, 5.857–24.591; *p <* 0.001) were materially unchanged from those in Model 1. Overall, Model 2 suggested possible but inconclusive effect heterogeneity according to BMI in the primary interaction analysis.

### 3.5 Primary BMI-stratified analyses based on model 2

BMI-stratified analyses based on Model 2 are presented in Table 4. In the lower-BMI stratum (<24 kg/m^2^; n = 209), the adjusted odds ratio for osteoporosis per 100 m increase in residential altitude was 0.986 (95% CI, 0.931–1.044; *p* = 0.620). In the higher-BMI stratum (≥24 kg/m^2^; n = 168), the corresponding estimate was 1.075 (95% CI, 0.999–1.157; *p* = 0.053).

**Table 4:** Association between residential altitude and osteoporosis stratified by BMI category.

BMI stratum	Model	Altitude (per +100 m) aOR (95% CI)	*p-*value for altitude	Notes
BMI < 24 kg/m^2^ (n = 209)	Primary (Model 2)	0.986 (0.931–1.044)	0.620	HU not adjusted
BMI ≥ 24 kg/m^2^ (n = 168)	Primary (Model 2)	1.075 (0.999–1.157)	**0.053**	HU not adjusted

Adjusted odds ratios (aORs) and 95% confidence intervals (CIs) for osteoporosis per 100 m higher residential altitude were estimated using multivariable binary logistic regression within each BMI stratum. BMI was stratified at 24.0 kg/m^2^. These stratified analyses were based on the primary interaction model (Model 2) and adjusted for age, sex, BMI (continuous within stratum), prior vertebral fracture, sodium, serum 25(OH)D, and albumin-corrected calcium. They are presented to aid interpretation of the primary interaction pattern and should be regarded as hypothesis-generating. Reported estimates are pooled across 20 multiply imputed datasets where applicable. *p-*values are two-sided. Bold values indicate statistically significant differences (two-sided test, *p* < 0.05).

**Figure 4:**
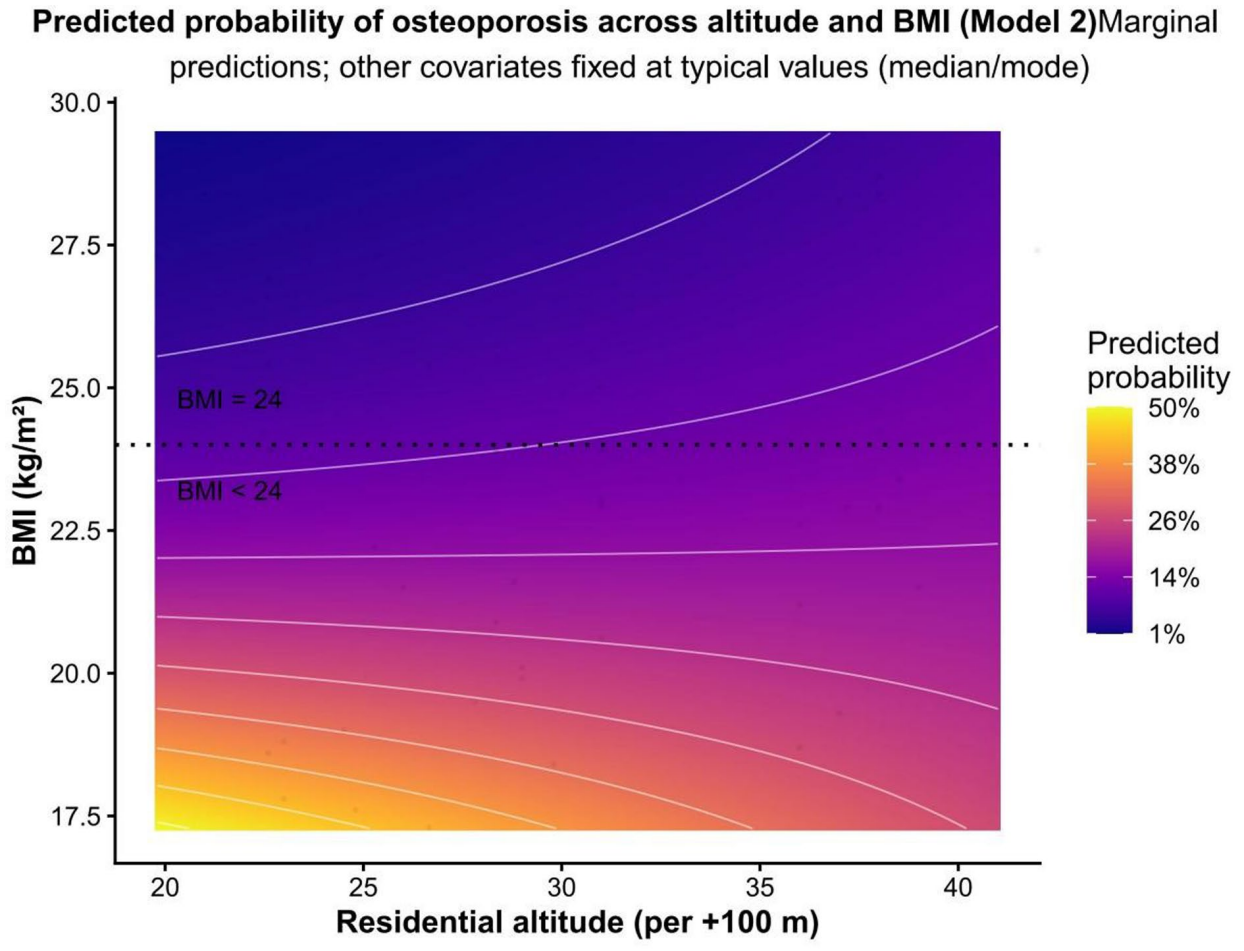
Predicted probability of osteoporosis across residential altitude and BMI (Model 2). The heatmap shows the adjusted model-based predicted probability of osteoporosis across residential altitude and BMI, based on Model 2. Predictions were generated with age, sex, prior vertebral fracture, serum sodium, and albumin-corrected calcium held constant, and serum 25(OH)D fixed at the sample median (12.76 ng/mL). BMI was limited to the 5th to 95th percentile range of the observed distribution. The plot highlights the altitude-dependent pattern of osteoporosis risk across BMI levels.

**Figure 5:**
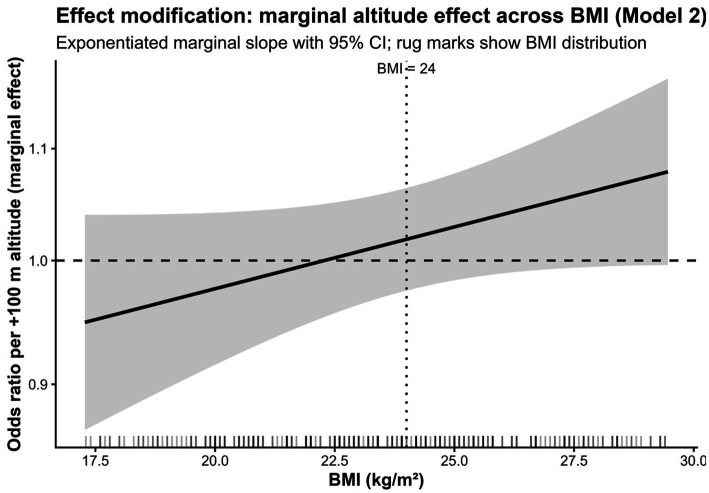
Conditional odds ratio for osteoporosis per 100 m higher residential altitude across BMI (Model 2). The plot displays the conditional odds ratio (OR) for osteoporosis per 100-m higher residential altitude across the BMI range, based on Model 2, which included interaction terms for residential altitude × BMI and residential altitude × 25(OH)D. Shaded areas represent 95% confidence intervals. BMI was restricted to the 5th to 95th percentile range of the observed sample, and serum 25(OH)D was fixed at the sample median value (12.76 ng/mL). The horizontal reference line denotes OR = 1, and the vertical dotted line marks BMI = 24 kg/m^2^.

These stratum-specific estimates were directionally divergent but imprecise and are presented only to aid interpretation of the borderline formal interaction result in Model 2. Because the altitude × BMI interaction did not meet the conventional two-sided 0.05 threshold in the primary model, the stratified results should be regarded as hypothesis-generating rather than confirmatory evidence of distinct subgroup-specific mechanisms. The model-based pattern is visualised in Figures 4, 5. Figure 4 shows the predicted probability surface across residential altitude and BMI. Figure 5 shows the conditional odds ratio for altitude as a function of BMI.

### 3.6 Exploratory HU-adjusted sensitivity analyses

Exploratory sensitivity analyses additionally adjusting for L1 trabecular HU were performed to assess whether the observed interaction pattern was materially altered after accounting for a proximal CT-derived skeletal phenotype. These HU-adjusted analyses yielded some strengthening of the altitude × BMI interaction estimate; however, because HU may represent a mediator-like skeletal phenotype closely related to the outcome, these findings were not considered primary and are reported in the Supplementary Tables S3, S4 and Supplementary Figure S3. The primary interpretation of BMI-related heterogeneity therefore remains based on Model 2.

## 4 Discussion

### 4.1 Main findings

In this hospital-based case–control study of long-term residents of the Qinghai–Tibetan Plateau, residential altitude was not independently associated with DXA-defined osteoporosis after multivariable adjustment. When effect modification was formally examined, the primary interaction model provided suggestive but imprecise evidence that the association between residential altitude and osteoporosis might vary according to BMI (altitude × BMI aOR 1.011, 95% CI 0.998–1.023; *p* = 0.087). No comparable interaction was observed for serum 25(OH)D. BMI-stratified estimates from the primary model suggested directionally different patterns, but these estimates were based on a borderline interaction test and relatively wide confidence intervals in relation to the observed effect size. Accordingly, they should be interpreted as hypothesis-generating rather than as definitive evidence of subgroup-specific biology.

Opportunistic CT-derived L1 trabecular attenuation was also strongly associated with osteoporosis status, supporting the potential utility of HU as a complementary skeletal phenotype in settings where access to DXA may be limited (43–45). Exploratory HU-adjusted sensitivity analyses, reported in Supplementary materials, are consistent with the possibility that the observed pattern may depend in part on proximal skeletal phenotype; however, these analyses do not alter the primary conclusion, which rests on Model 2. Overall, the data were more compatible with possible phenotype-dependent heterogeneity than with a uniform skeletal effect of altitude. However, this interpretation remains provisional because the main effect was null and the interaction evidence in the primary model was only borderline significant.

### 4.2 Prior evidence and how this study addresses prior inconsistencies

Previous studies on altitude and bone health have reported heterogeneous findings, including adverse skeletal profiles, heterogeneous findings, and apparently preserved bone status in some high-altitude populations (4, 10, 13, 20). These inconsistencies may reflect differences in study design, exposure definition, population adaptation, body composition, and confounding structure rather than direct contradictions among studies.

The present study contributes to the literature in two important ways. First, residential altitude was evaluated as a continuous exposure within a single plateau region characterized by substantial variation in elevation. Second, the study explicitly assessed whether the association between altitude and osteoporosis differed according to host phenotype, particularly BMI. Rather than supporting a consistent overall effect of altitude, the findings suggest that some of the inconsistency in prior studies may be explained by heterogeneity in skeletal responses across individuals. Although this interpretation remains exploratory, it offers a coherent framework for understanding why previous studies have reported differing results.

### 4.3 Why high-altitude residence may exacerbate osteoporosis risk among higher-BMI individuals

One possible explanation for the directionally positive altitude-associated pattern observed among participants with higher BMI is that the net skeletal effects of chronic altitude exposure may vary according to metabolic context. In lowland settings, higher BMI is often associated with greater bone mass, partly through mechanical loading and peripheral oestrogen production. However, excess adiposity is also associated with adipose-tissue hypoxia, chronic low-grade inflammation, altered adipokine signalling, and changes in the marrow microenvironment. These factors may adversely affect bone remodelling (46–49). Under chronic hypobaric hypoxia, these adverse metabolic features may become more important and may offset some of the skeletal advantages traditionally attributed to higher BMI.

These mechanisms are biologically plausible, but they were not directly evaluated in the present study. Body composition, inflammatory biomarkers, adipokines, and bone-turnover markers were not assessed. Therefore, the mechanistic interpretation should remain cautious. The current data support the possibility of BMI-related heterogeneity, but they do not establish the specific biological pathways through which this heterogeneity may arise.

### 4.4 Interpreting the potential protective trend among lower-BMI individuals

Among participants with BMI < 24 kg/m^2^, the altitude-associated estimates were directionally lower in the primary model and appeared more pronounced only in the HU-adjusted sensitivity analysis. Several explanations may account for this pattern. Individuals with lower BMI may have a lower burden of adiposity-related inflammation and metabolic dysfunction, which could reduce susceptibility to hypoxia-related skeletal stress (46, 48). Long-term residence at high altitude may also select for physiologically resilient individuals with more favourable metabolic or vascular adaptation profiles. In addition, unmeasured differences in physical activity, diet, frailty, or survivorship may have contributed to the observed pattern.

This finding should not be overinterpreted. The primary interaction model did not meet the conventional significance threshold of 0.05, and the lower-BMI pattern became more apparent only after additional adjustment for HU. Accordingly, the findings in the lower-BMI subgroup should be regarded as hypothesis-generating rather than as evidence that altitude is protective in leaner plateau residents. Confirmation in larger prospective cohorts will be necessary before any protective interpretation can be made.

### 4.5 Opportunistic CT-derived HU as both a methodological asset and a mechanistic clue

We observed a strong inverse association between L1 trabecular attenuation and DXA-defined osteoporosis. This finding is consistent with the growing literature supporting opportunistic vertebral HU as a practical adjunct for skeletal risk assessment (43, 50). This finding is particularly relevant in plateau settings, where access to DXA may be limited and routine CT examinations may provide an opportunity for low-cost secondary osteoporosis screening.

In supplementary exploratory sensitivity analyses, adjustment for L1 trabecular HU altered the estimated altitude × BMI interaction pattern. However, the role of HU in this context should be interpreted with caution. HU is closely related to skeletal status and may represent a proximal skeletal phenotype or mediator-related variable rather than a conventional confounder. For this reason, the strengthening of the altitude × BMI interaction after HU adjustment should not be interpreted as a more valid estimate of the total altitude-associated effect and may reflect over-adjustment. Accordingly, the HU-adjusted model is best regarded as a supplementary exploratory sensitivity analysis rather than a primary inferential model. These findings suggest that the trabecular skeletal phenotype may be intertwined with the observed pattern of heterogeneity. Longitudinal studies with repeated HU and DXA measurements will be needed to clarify whether HU acts primarily as a proxy for confounding, a mediator-related phenotype, or both.

### 4.6 Reconsidering the 25(OH)D effect-modification hypothesis

Serum 25(OH)D did not appear to modify the association between residential altitude and osteoporosis in the present study. This negative interaction finding should be interpreted cautiously and should not be regarded as definitive evidence that no biological interplay exists. One possible explanation is the limited variation in vitamin D concentrations, which were generally low across the cohort. Another is that a single circulating 25(OH)D measurement may not adequately reflect long-term vitamin D physiology in plateau environments, where sunlight exposure, behavioural patterns, supplementation, diet, and adaptive responses may vary over time (51–53).

From a clinical perspective, the generally low 25(OH)D levels remain relevant, even though no interaction was detected. Future studies should reassess this question in cohorts with a wider range of vitamin D levels and more detailed characterization of supplementation, diet, and sun-exposure behaviour.

### 4.7 Limitations

Several limitations should be acknowledged. First, this was a single-centre, hospital-based retrospective case–control study using an extreme-phenotype design. Controls were drawn mainly from health-examination and opportunistic imaging pathways, whereas cases were more often identified through clinical referral pathways; such differential sampling may have introduced selection bias. In addition, extreme-phenotype sampling improves contrast but limits transportability, so the observed odds ratios should not be interpreted as community-level estimates or absolute-risk analogues. Second, residential altitude was derived from geocoded long-term address records and may not fully capture cumulative lifetime exposure, migration history, or critical exposure windows relevant to bone accrual and loss. Third, although multiple imputation was applied to missing laboratory covariates, residual confounding cannot be excluded. Data on diet, physical activity, smoking intensity, cumulative glucocorticoid exposure, body composition, bone-turnover markers, and other relevant clinical factors were not comprehensively available. In addition, self-reported ethnicity was not routinely recorded in the retrospective dataset and therefore could not be incorporated into the analyses. Given the substantial ethnic diversity of Qinghai, unmeasured differences in ethnic composition and ethnicity-associated contextual factors, such as residential patterns, diet, lifestyle, socioeconomic conditions, ultraviolet exposure, and access to healthcare, may have contributed to residual confounding. Therefore, future studies with prospectively collected ethnicity and related contextual information are needed to better disentangle the effects of residential altitude from population-structure and lifestyle-related factors.

Fourth, given the modest number of controls (n = 153) relative to cases and the limited number of events within BMI subgroups, the study may have insufficient statistical power to detect modest interaction effects. Consequently, the borderline *p*-value of 0.087 for the primary altitude × BMI interaction could reflect a Type II error. The observed BMI-related heterogeneity should therefore be interpreted as suggestive and hypothesis-generating rather than confirmatory. Finally, although HU-adjusted analyses provided additional exploratory signals in sensitivity analyses, HU is closely related to the skeletal outcome and may lie near the causal pathway. Accordingly, HU-adjusted estimates should not be interpreted as the primary total-effect results. Prospective multicentre studies with more detailed exposure histories, repeated skeletal phenotyping, and fracture follow-up are needed to confirm these findings. Moreover, occupational altitude exposure was not captured in our dataset. Therefore, cumulative altitude exposure may be misclassified if participants’ workplace altitude differed from their residential altitude, which could introduce residual confounding.

### 4.8 Public health implications

From a public health perspective, the present findings may be relevant for osteoporosis screening in remote high-altitude regions, where formal DXA-based assessment is not always readily accessible. Although residential altitude was not independently associated with osteoporosis after adjustment, the observed possibility of BMI-related heterogeneity suggests that easily obtainable host characteristics may help refine risk stratification in plateau populations. In such settings, opportunistic use of routine CT imaging may represent a pragmatic supplementary pathway for identifying individuals who warrant further skeletal evaluation, particularly when dedicated osteoporosis screening resources are limited. At the same time, these implications should remain cautious because the current study was exploratory, hospital-based, and not designed to establish screening thresholds or policy recommendations. Larger population-based studies are needed to determine whether altitude-related bone health patterns can be translated into equitable and effective public health screening strategies for underserved plateau communities.

## 5 Conclusion

In this single-centre, hospital-based retrospective case–control study of long-term residents of the Qinghai–Tibetan Plateau, residential altitude was not independently associated with DXA-defined osteoporosis after multivariable adjustment. The data provided suggestive but inconclusive evidence that the association between residential altitude and osteoporosis may vary according to BMI. In the primary interaction model, directionally different patterns were observed across BMI strata, although these estimates were imprecise. These findings should therefore be regarded as exploratory and hypothesis-generating rather than confirmatory.

Opportunistic CT-derived vertebral trabecular attenuation was strongly associated with osteoporosis status and may represent a useful complementary skeletal phenotype in settings where access to DXA is limited. While these findings require confirmation in larger prospective studies, they may help inform future public health approaches to osteoporosis risk stratification and opportunistic screening in resource-limited high-altitude populations.

## Data Availability

The datasets generated and/or analyzed during the current study are not publicly available due to patient privacy and confidentiality agreements but are available from the corresponding author on reasonable request. Requests to access the datasets should be directed to ZL, 18765610018@163.com.

## References

[1] GBD 2021 Low Bone Mineral Density Collaborators. The global, regional, and national burden attributable to low bone mineral density, 1990-2020: an analysis of a modifiable risk factor from the Global Burden of Disease Study 2021. Integr Comp Biol. (2025) 7:e873–e894. doi: 10.1016/S2665-9913(25)00105-5 PMC12623303 40972625

[2] Wu AM Bisignano C James SL Abady GG. Global, regional, and national burden of bone fractures in 204 countries and territories, 1990-2019: a systematic analysis from the global burden of disease study 2019. Lancet Healthy Longev. (2021) 2:e580–92. doi: 10.1016/S2666-7568(21)00172-0, 34723233 PMC8547262

[3] Wang S Zhang F Guo Y Zhang C Zhong Y Hao D . Exposure to high altitude is associated with an elevated risk of hip fracture: a retrospective cohort study using data from the CHARLS. BMC Geriatr. (2025) 25:884. doi: 10.1186/s12877-025-06492-6, 41214560 PMC12604388

[4] Brent MB. A review of the skeletal effects of exposure to high altitude and potential mechanisms for hypobaric hypoxia-induced bone loss. Bone. (2022) 154:116258. doi: 10.1016/j.bone.2021.116258, 34781048

[5] Xu Y Hua Q Chen W Gong J Meng X Wang T . Human adaptation to high altitude: acclimatization and reversibility of haemodynamics. Natl Sci Rev. (2025) 12. doi: 10.1093/nsr/nwaf203, 40585565 PMC12202200

[6] Beall CM. Andean, Tibetan, and Ethiopian patterns of adaptation to high-altitude hypoxia. Integr Comp Biol. (2006) 46:18–24. doi: 10.1093/icb/icj004, 21672719

[7] Meng X Wielockx B Rauner M Bozec A. Hypoxia-inducible factors regulate osteoclasts in health and disease. Front Cell Dev Biol. (2021) 9:658893. doi: 10.3389/fcell.2021.658893, 33816509 PMC8014084

[8] Qiao Y Zheng H Cheng R Rong L Guo J Li G . A review of the relationship between gut microbiota and osteoporosis in high altitude environments. J Health Popul Nutr. (2025) 44:241. doi: 10.1186/s41043-025-00994-0, 40624713 PMC12235887

[9] Babu L Ghosh D. Looking at mountains: role of sustained hypoxia in regulating bone mineral homeostasis in relation to Wnt pathway and estrogen. Clin Rev Bone Miner Metab. (2022) 20:18–36. doi: 10.1007/s12018-022-09283-4

[10] Zuo H Zheng T Wu K Yang T Wang L Nima Q . High-altitude exposure decreases bone mineral density and its relationship with gut microbiota: results from the China multi-ethnic cohort (CMEC) study. Environ Res. (2022) 215:114206. doi: 10.1016/j.envres.2022.114206, 36058270

[11] Xu R Li T Wang Z Wang H Sun M Xie J . Association among lean mass, gut microbiome alterations and bone mineral density in high-altitude. Clin Nutr. (2025) 51:18–27. doi: 10.1016/j.clnu.2025.05.018, 40516324

[12] Babu LK Shaw S Ghosh D. Bone mineral metabolism and different indices of skeletal health of Ladakhi women living at high altitude. Osteoporos Sarcopenia. (2023) 9:131–6. doi: 10.1016/j.afos.2023.11.001, 38374823 PMC10874723

[13] Tanaka H Minowa K Satoh T Koike T. Bone atrophy at high altitude. J Bone Miner Metab. (1992) 10:31–6. doi: 10.1007/bf02383459

[14] Turcotte AF O'Connor S Morin SN Gibbs JC Willie BM Jean S . Association between obesity and risk of fracture, bone mineral density and bone quality in adults: a systematic review and meta-analysis. PLoS One. (2021) 16:e0252487. doi: 10.1371/journal.pone.0252487 34101735 PMC8186797

[15] Cao JJ. Effects of obesity on bone metabolism. J Orthop Surg Res. (2011) 6:30. doi: 10.1186/1749-799x-6-30, 21676245 PMC3141563

[16] Lee KC Lanyon LE. Mechanical loading influences bone mass through estrogen receptor alpha. Exerc Sport Sci Rev. (2004) 32:64–8. doi: 10.1097/00003677-200404000-00005, 15064650

[17] Greco EA Lenzi A Migliaccio S. The obesity of bone. Ther Adv Endocrinol Metab. (2015) 6:273–86. doi: 10.1177/2042018815611004, 26623005 PMC4647134

[18] Wood IS de Heredia FP Wang B Trayhurn P. Cellular hypoxia and adipose tissue dysfunction in obesity. Proc Nutr Soc. (2009) 68:370–7. doi: 10.1017/S0029665109990206, 19698203

[19] Forte YS Renovato-Martins M Barja-Fidalgo C. Cellular and molecular mechanisms associating obesity to bone loss. Cells. (2023) 12:521. doi: 10.3390/cells12040521, 36831188 PMC9954309

[20] Zhong H Zhou Y Wang P Jia Q Wan Y Xiong H. Influencing factors of bone mass abnormalities among postmenopausal women in Tibet, China. BMC Public Health. (2023) 23:2100. doi: 10.1186/s12889-023-17015-6, 37880645 PMC10601267

[21] Yang R Ma Q Zhang X Zhao Q Zeng S Yan H . A study on the prevalence of osteoporosis in people with different altitudes in Sichuan, China. Clin Interv Aging. (2024) 19:1819–28. doi: 10.2147/CIA.S478020, 39525876 PMC11549880

[22] Norsang G Ma L Dahlback A Zhuoma C Tsoja W Porojnicu A . The vitamin D status among Tibetans. Photochem Photobiol. (2009) 85:1028–31. doi: 10.1111/j.1751-1097.2009.00552.x, 19508646

[23] Mata-Greenwood E Westenburg HCA Zamudio S Illsley NP Zhang L. Decreased vitamin D levels and altered placental vitamin D gene expression at high altitude: role of genetic ancestry. Int J Mol Sci. (2023) 24:389. doi: 10.3390/ijms24043389, 36834800 PMC9967090

[24] Gregson CL Armstrong DJ Avgerinou C Bowden J Cooper C Douglas L . The 2024 UK clinical guideline for the prevention and treatment of osteoporosis. Arch Osteoporos. (2025) 20:119. doi: 10.1007/s11657-025-01588-3, 40921943 PMC12417299

[25] Harvey NC Al-Daghri N Beaudart C Brandi ML Burlet N Campusano C . Barriers and solutions for global access to osteoporosis management: a position paper from the international osteoporosis foundation. Osteoporos Int. (2025) 36:1495–507. doi: 10.1007/s00198-025-07628-5, 40844608 PMC12460584

[26] Stephanus AD Ramos SCL Netto OS de Carvalho LSF Campos-Staffico AM. Fracture risk assessment tool-based screening for osteoporosis in older adults in resource-limited settings. J Clin Densitom. (2024) 27:101494. doi: 10.1016/j.jocd.2024.101494, 38677082

[27] Pickhardt PJ Pooler BD Lauder T del Rio AM Bruce RJ Binkley N. Opportunistic screening for osteoporosis using abdominal computed tomography scans obtained for other indications. Ann Intern Med. (2013) 158:588–95. doi: 10.7326/0003-4819-158-8-201304160-00003, 23588747 PMC3736840

[28] Zhu Y Triphuridet N Yip R Becker BJ Wang Y Yankelevitz DF . Opportunistic CT screening of osteoporosis on thoracic and lumbar spine: a meta-analysis. Clin Imaging. (2021) 80:382–90. doi: 10.1016/j.clinimag.2021.08.005, 34530357

[29] Lee SJ Binkley N Lubner MG Bruce RJ Ziemlewicz TJ Pickhardt PJ. Opportunistic screening for osteoporosis using the sagittal reconstruction from routine abdominal CT for combined assessment of vertebral fractures and density. Osteoporos Int. (2016) 27:1131–6. doi: 10.1007/s00198-015-3318-4, 26419470

[30] Vadera S Osborne T Shah V Stephenson JA. Opportunistic screening for osteoporosis by abdominal CT in a British population. Insights Imaging. (2023) 14:57. doi: 10.1186/s13244-023-01400-1, 37005941 PMC10067782

[31] Chen L Fouladi RT. Correcting bias in extreme groups design using a missing data approach. Psychol Methods. (2024) 29:1123–31. doi: 10.1037/met0000508, 35849371

[32] Preacher KJ Rucker DD MacCallum RC Nicewander WA. Use of the extreme groups approach: a critical reexamination and new recommendations. Psychol Methods. (2005) 10:178–92. doi: 10.1037/1082-989X.10.2.178, 15998176

[33] Kanis JA. Assessment of fracture risk and its application to screening for postmenopausal osteoporosis: synopsis of a WHO report. WHO study group. Osteoporos Int. (1994) 4:368–81. doi: 10.1007/BF01622200, 7696835

[34] Shuhart C Cheung A Gill R Gani L Goel H Szalat A. Executive summary of the 2023 adult position development conference of the International Society for Clinical Densitometry: DXA reporting, follow-up BMD testing and trabecular bone score application and reporting. J Clin Densitom. (2024) 27:101435. doi: 10.1016/j.jocd.2023.101435, 38007332

[35] Wildman RP Gu D Reynolds K Duan X He J. Appropriate body mass index and waist circumference cutoffs for categorization of overweight and central adiposity among Chinese adults. Am J Clin Nutr. (2004) 80:1129–36. doi: 10.1093/ajcn/80.5.1129, 15531658

[36] Acevedo JBH Lenchik L Weaver AA Boutin RD Wuertzer S. Opportunistic screening of bone fragility using computed tomography. Semin Musculoskelet Radiol. (2024) 28:620–7. doi: 10.1055/s-0044-1788816, 39406224 PMC12151326

[37] Koo TK Li MY. A guideline of selecting and reporting Intraclass correlation coefficients for reliability research. J Chiropr Med. (2016) 15:155–63. doi: 10.1016/j.jcm.2016.02.012, 27330520 PMC4913118

[38] Genant HK Wu CY van Kuijk C Nevitt MC. Vertebral fracture assessment using a semiquantitative technique. J Bone Miner Res. (1993) 8:1137–48. doi: 10.1002/jbmr.5650080915, 8237484

[39] Holick MF Binkley NC Bischoff-Ferrari HA Gordon CM Hanley DA Heaney RP . Evaluation, treatment, and prevention of vitamin D deficiency: an Endocrine Society clinical practice guideline. J Clin Endocrinol Metab. (2011) 96:1911–30. doi: 10.1210/jc.2011-0385, 21646368

[40] Sterne JA White IR Carlin JB Spratt M Royston P Kenward MG . Multiple imputation for missing data in epidemiological and clinical research: potential and pitfalls. BMJ. (2009) 338:b2393. doi: 10.1136/bmj.b2393, 19564179 PMC2714692

[41] White IR Royston P Wood AM. Multiple imputation using chained equations: issues and guidance for practice. Stat Med. (2011) 30:377–99. doi: 10.1002/sim.4067, 21225900

[42] Marshall A Altman DG Holder RL Royston P. Combining estimates of interest in prognostic modelling studies after multiple imputation: current practice and guidelines. BMC Med Res Methodol. (2009) 9:57. doi: 10.1186/1471-2288-9-57, 19638200 PMC2727536

[43] Lee S Chung CK Oh SH Park SB. Correlation between bone mineral density measured by dual-energy X-ray absorptiometry and Hounsfield units measured by diagnostic CT in lumbar spine. J Korean Neurosurg Soc. (2013) 54:384–9. doi: 10.3340/jkns.2013.54.5.384, 24379944 PMC3873350

[44] Pickhardt PJ Lauder T Pooler BD Muñoz Del Rio A Rosas H Bruce RJ . Effect of IV contrast on lumbar trabecular attenuation at routine abdominal CT: correlation with DXA and implications for opportunistic osteoporosis screening. Osteoporos Int. (2016) 27:147–52. doi: 10.1007/s00198-015-3224-9, 26153046

[45] Yang G Wang H Wu Z Shi Y Zhao Y. Prediction of osteoporosis and osteopenia by routine computed tomography of the lumbar spine in different regions of interest. J Orthop Surg Res. (2022) 17:454. doi: 10.1186/s13018-022-03348-2, 36243720 PMC9571421

[46] Rinonapoli G Pace V Ruggiero C Ceccarini P Bisaccia M Meccariello L . Obesity and bone: a complex relationship. Int J Mol Sci. (2021) 22:13662. doi: 10.3390/ijms222413662, 34948466 PMC8706946

[47] Huynh PM Wang F An YA. Hypoxia signaling in the adipose tissue. J Mol Cell Biol. (2024) 16:mjae039. doi: 10.1093/jmcb/mjae039, 39363240 PMC11892559

[48] Benova A Tencerova M. Obesity-induced changes in bone marrow homeostasis. Front Endocrinol. (2020) 11:294. doi: 10.3389/fendo.2020.00294, 32477271 PMC7235195

[49] Deepika F Bathina S Armamento-Villareal R. Novel adipokines and their role in bone metabolism: a narrative review. Biomedicine. (2023) 11:644. doi: 10.3390/biomedicines11020644, 36831180 PMC9953715

[50] Boutin RD Lenchik L. Value-added opportunistic CT: insights into osteoporosis and sarcopenia. AJR Am J Roentgenol. (2020) 215:582–94. doi: 10.2214/AJR.20.22874, 32755187

[51] Giustina A Bilezikian JP Adler RA Banfi G Bikle DD Binkley NC . Consensus statement on vitamin D status assessment and supplementation: whys, Whens, and Hows. Endocr Rev. (2024) 45:625–54. doi: 10.1210/endrev/bnae009, 38676447 PMC11405507

[52] Mazahery H Von Hurst PR. Factors affecting 25-Hydroxyvitamin D concentration in response to vitamin D supplementation. Nutrients. (2015) 7:5111–42. doi: 10.3390/nu7075111, 26121531 PMC4516990

[53] McKibben RA Zhao D Lutsey PL Schneider ALC Guallar E Mosley TH . Factors associated with change in 25-hydroxyvitamin D levels over longitudinal follow-up in the ARIC study. J Clin Endocrinol Metab. (2016) 101:33–43. doi: 10.1210/jc.2015-1711, 26509869 PMC4701839

