# Supplementary material for: Residential altitude was not independently associated with osteoporosis among long-term Qinghai–Tibetan Plateau residents
Source: Front Public Health. 2026 May 20;14:1844263. doi: 10.3389/fpubh.2026.1844263 (PMC13229694; doi:10.3389/fpubh.2026.1844263)
Supplement: Supplementary file 1 [file Supplementary_file_1.docx]

**Supplementary Table S1. Missing-data pattern and multiple imputation summary**

| **Variable** | **Analytic role** | **Missing n**  **(%)** | **Imputed** | **Included in imputation model** | **Notes** |
| --- | --- | --- | --- | --- | --- |
| Age | Core covariate | 0 (0.0) | No | Yes | Complete variable |
| Sex | Core covariate | 0 (0.0) | No | Yes | Complete variable |
| BMI | Core covariate / effect modifier | 0 (0.0) | No | Yes | Complete variable |
| Residential altitude | Primary exposure | 0 (0.0) | No | Yes | Complete variable |
| DXA-defined osteoporosis status | Outcome | 0 (0.0) | No | Yes | Complete variable |
| Prior vertebral fracture | Clinical covariate | 0 (0.0) | No | Yes | Complete variable |
| Opportunistic CT trabecular attenuation (L1 HU) | Sensitivity-model covariate | 0 (0.0) | No | Yes | Complete variable |
| Corrected calcium | Model covariate | 52 (13.8) | Yes | Yes | Imputed by MICE |
| 25(OH)D | Model covariate | 7 (1.9) | Yes | Yes | Imputed by MICE |
| Potassium | Candidate covariate | 13 (3.4) | Yes | Yes | Imputed by MICE |
| Sodium | Model covariate | 13 (3.4) | Yes | Yes | Imputed by MICE |
| Magnesium | Candidate covariate | 69 (18.3) | Yes | Yes | Imputed by MICE |
| Phosphate | Candidate covariate | 64 (17.0) | Yes | Yes | Imputed by MICE |
| Chloride | Candidate covariate | 13 (3.4) | Yes | Yes | Imputed by MICE |

**Notes:** Missingness was observed primarily in laboratory variables, whereas age, sex, BMI, residential altitude, DXA-defined osteoporosis status, prior vertebral fracture history, and opportunistic CT trabecular attenuation (L1 HU) were complete. Missing values were handled using multiple imputation by chained equations (MICE), generating twenty imputed datasets. Regression coefficients and standard errors were pooled using Rubin’s rules. Complete variables were not imputed but were included in the imputation model as predictors where appropriate. Variables designated as “model covariates” were included in the primary multivariable regression models, whereas “candidate covariates” were included in the imputation framework and considered during model development.

**Supplementary Figure S1. Bland–Altman plot of inter-reader agreement for L1 trabecular HU measurements.**

The x-axis shows the mean HU value of the two readers, and the y-axis shows the inter-reader difference (Reader 1 − Reader 2). The central horizontal line indicates the mean difference (bias), and the upper and lower horizontal lines indicate the 95% limits of agreement (bias ± 1.96 SD).

**Supplementary Table S2. Inter-reader reliability for L1 trabecular HU measurements**

| **Measure** | **Value** |
| --- | --- |
| Number of examinations | 377 |
| ICC model | Two-way random-effects, absolute agreement, single measure |
| ICC | 0.991 |
| 95% CI | 0.988–0.993 |
| P value | <0.001 |
| Interpretation | Excellent agreement |

**Notes:** Inter-reader reliability was assessed using a two-way random-effects intraclass correlation coefficient (ICC) for absolute agreement. The single-measure ICC was 0.991 (95% CI 0.988–0.993; P < 0.001), indicating excellent agreement between the two readers.

**Supplementary Figure S2. Representative example of vertebral attenuation measurement on opportunistic non-contrast CT.**


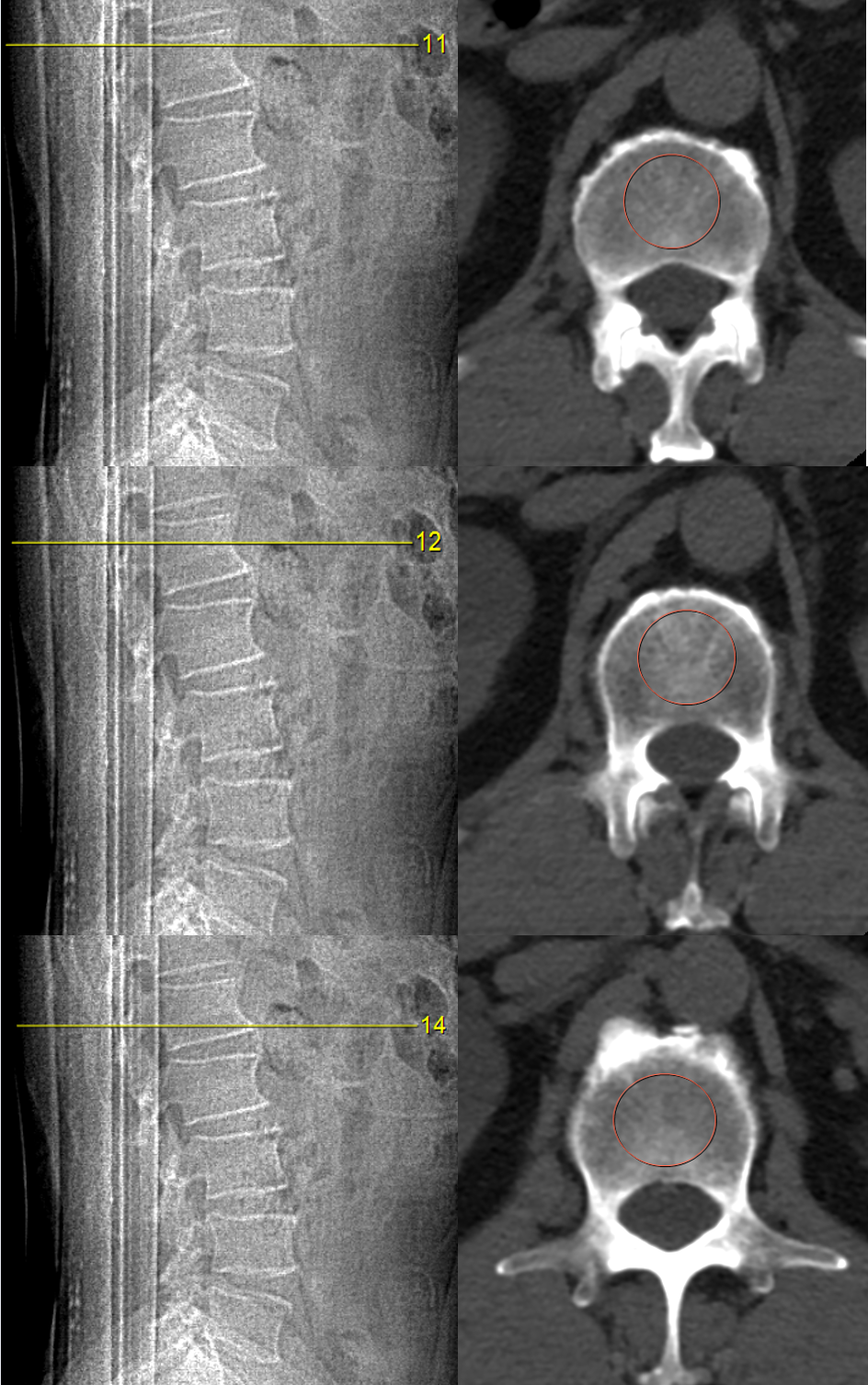


The left panels show the sagittal scout image used to identify three axial measurement levels within the L1 vertebral body. The right panels show oval regions of interest placed in the trabecular bone on three axial slices: just inferior to the superior endplate, at the mid-vertebral level, and just superior to the inferior endplate. Regions of interest were positioned to avoid the cortical shell, the basivertebral venous plexus, focal sclerosis, and other visible focal abnormalities. The mean attenuation across the three slices was used as the final vertebral attenuation value in Hounsfield units (HU).

**Supplementary Table S3. Multivariable logistic regression models for osteoporosis（Model 3）**

| **Table S3 \| Multivariable logistic regression models for osteoporosis** | | | |
| --- | --- | --- | --- |
| **Predictor** | **Coding/unit** | **Model 3 aOR (95% CI)** | **P value** |
| **Main effects and covariates** | | | |
| Age | per +1 year | 0.956  (0.902 – 1.014) | **0.134** |
| Female sex | female vs male | 11.032  (3.486 –34.915) | **<0.001** |
| BMI | per +1 kg/m² | 0.702  (0.603 – 0.817) | **0.001** |
| Residential altitude | per +100 m | 0.953  (0.888 – 1.023) | 0.185 |
| Prior vertebral fracture | yes vs no | 4.476  (1.424 – 14.068) | **0.010** |
| Sodium | per +1 mmol/L | 1.121  (0.959 – 1.309) | 0.152 |
| 25(OH)D | per +1 ng/mL | 0.955  (0.897 – 1.016) | 0.146 |
| Corrected calcium | per +1 mmol/L | 3.039  (0.158 – 58.386) | 0.461 |
| L1 HU | per +10 HU | 0.382  (0.290 – 0.504) | **<0.001** |
| **Interaction terms** | | | |
| Altitude × BMI | interaction term | 1.023  (1.003–1.042) | **0.021** |
| Altitude × 25(OH)D | interaction term | 0.998  (0.989 – 1.006) | 0.590 |

**Notes:** Adjusted odds ratios (aORs) and 95% confidence intervals (CIs) were estimated using multivariable binary logistic regression with osteoporosis status (yes/no) as the outcome. This supplementary exploratory sensitivity model (Model 3) included age, sex, BMI, residential altitude, prior vertebral fracture, sodium, serum 25(OH)D, albumin-corrected calcium, opportunistic CT-derived L1 trabecular HU, and the prespecified interaction terms residential altitude × BMI and residential altitude × 25(OH)D. Continuous predictors were modelled per unit increase as indicated in the “Coding/unit” column. Residential altitude was scaled per 100 m and L1 trabecular HU per 10 HU. To reduce collinearity, continuous variables entering interaction terms were mean-centred before construction of cross-product terms. Reported estimates are pooled across twenty multiply imputed datasets where applicable. P values are two-sided Wald test P values for the corresponding coefficients; for the rows “Altitude × BMI” and “Altitude × 25(OH)D”, the P values represent formal tests of multiplicative interaction.

**Supplementary Table S4. Association between residential altitude and osteoporosis stratified by BMI category**

| **Table S4 \| Association between altitude and osteoporosis stratified by BMI** | | | | |
| --- | --- | --- | --- | --- |
| **BMI stratum** | **Model** | **Altitude (per +100 m) aOR (95% CI)** | **P value for altitude** | **Notes** |
| BMI < 24 kg/m² (n = 209) | Sensitivity (Model 3) | 0.871 (0.779 – 0.973) | **0.014** | +HU adjusted |
| BMI ≥ 24 kg/m² (n = 168) | Sensitivity (Model 3) | 1.056 (0.952 – 1.173) | 0.302 | +HU adjusted |

**Notes:** Adjusted odds ratios (aORs) and 95% confidence intervals (CIs) for osteoporosis per 100 m higher residential altitude were estimated using multivariable binary logistic regression within each BMI stratum. BMI was stratified at 24.0 kg/m². These supplementary sensitivity analyses were based on Model 3 and additionally adjusted for opportunistic CT-derived L1 trabecular HU (per 10 HU), along with age, sex, BMI (continuous within stratum), prior vertebral fracture, sodium, serum 25(OH)D, and albumin-corrected calcium. Reported estimates are pooled across twenty multiply imputed datasets where applicable. P values are two-sided.

**Supplementary Figure S3. Marginal odds ratios for osteoporosis per 100 m higher residential altitude across BMI, with and without HU adjustment.**

**
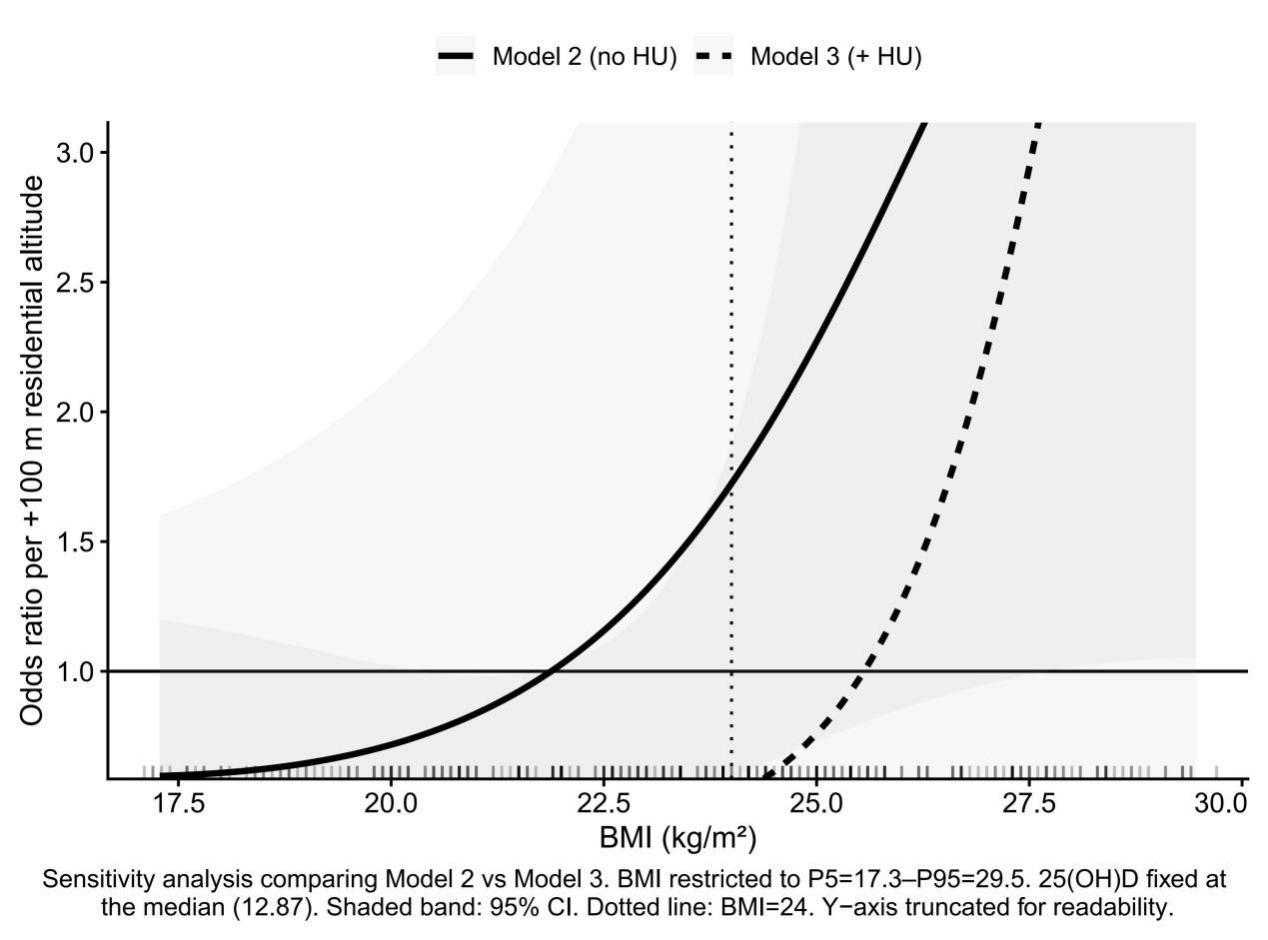
**

Shown are model-based marginal odds ratios (ORs) for osteoporosis per 100 m higher residential altitude across BMI derived from Model 2 (without HU adjustment) and Model 3 (with additional adjustment for L1 trabecular HU, per 10 HU). Both models included the residential altitude × BMI and residential altitude × 25(OH)D interaction terms and adjusted for age, sex, prior vertebral fracture, sodium, and albumin-corrected calcium, with serum 25(OH)D fixed at the sample median (12.76 ng/mL). BMI values were restricted to the 5th–95th percentile range. The vertical dotted line denotes BMI = 24 kg/m², and the horizontal reference line denotes OR = 1.
